# Genetic ablation of dynactin p150^Glued^ in postnatal neurons causes preferential degeneration of spinal motor neurons in aged mice

**DOI:** 10.1186/s13024-018-0242-z

**Published:** 2018-03-01

**Authors:** Jia Yu, Chen Lai, Hoon Shim, Chengsong Xie, Lixin Sun, Cai-Xia Long, Jinhui Ding, Yan Li, Huaibin Cai

**Affiliations:** 1 Institute for Geriatrics and Rehabilitation, Beijing Geriatric Hospital, Beijing University of Chinese Medicine, Beijing, 100095 People’s Republic of China; 2 Transgenic Section, Laboratory of Neurogenetics, National Institute on Aging, National Institutes of Health, Building 35, Room 1A112, MSC 3707, 35 Convent Drive, Bethesda, MD 20892–3707 USA; 3 Computational Biology Core, Laboratory of Neurogenetics, National Institute on Aging, National Institutes of Health, Bethesda, MD 20892 USA; 4 NINDS Protein/Peptide Sequencing Facility, National Institute of Neurological Disorders and Stroke, National Institutes of Health, Bethesda, MD 20892 USA; 5 Present address: Symptom Management Branch, National Institute of Nursing Research, National Institutes of Health, Bethesda, MD 20892 USA; 6 Department of Anesthesiology, Albert Einstein College of Medicine, Bronx, New York, 10467 USA; 7 Dendrite Morphogenesis and Plasticity Unit, National Institute of Neurological Disorders and Stroke, National Institutes of Health, Bethesda, MD 20892 USA

**Keywords:** Dynactin p150^Glued^, Dynein, Microtubule binding domain, Motor neuron, Neurodegeneration, Autophagy, Lysosome, Glutamate receptor, Excitotoxicity

## Abstract

**Background:**

Dynactin p150^Glued^, the largest subunit of the dynactin macromolecular complex, binds to both microtubules and tubulin dimers through the N-terminal cytoskeleton-associated protein and glycine-rich (CAP-Gly) and basic domains, and serves as an anti-catastrophe factor in stabilizing microtubules in neurons. P150^Glued^ also initiates dynein-mediated axonal retrograde transport. Multiple missense mutations at the CAP-Gly domain of p150^Glued^ are associated with motor neuron diseases and other neurodegenerative disorders, further supporting the importance of microtubule domains (MTBDs) in p150^Glued^ functions. However, most functional studies were performed in vitro. Whether p150^Glued^ is required for neuronal function and survival in vivo is unknown.

**Methods:**

Using Cre-loxP genetic manipulation, we first generated a line of p150^Glued^ knock-in mice by inserting two *LoxP* sites flanking the MTBD-coding exons 2 to 4 of p150^Glued^–encoding *Dctn1* gene (*Dctn1*^LoxP/^), and then crossbred the resulting *Dctn1*^LoxP/^ mice with *Thy1-Cre* mice to generate the bigenic p150^Glued^ (*Dctn1*^LoxP/LoxP^; *Thy1-Cre*) conditional knockout (cKO) mice for the downstream motor behavioral and neuropathological studies.

**Results:**

P150^Glued^ expression was completely abolished in Cre-expressing postnatal neurons, including corticospinal motor neurons (CSMNs) and spinal motor neurons (SMNs), while the MTBD–truncated forms remained. P150^Glued^ ablation did not affect the formation of dynein/dynactin complex in neurons. The p150^Glued^ cKO mice did not show any obvious developmental phenotypes, but exhibited impairments in motor coordination and rearing after 12 months of age. Around 20% loss of SMNs was found in the lumbar spinal cord of 18-month-old cKO mice, in company with increased gliosis, neuromuscular junction (NMJ) disintegration and muscle atrophy. By contrast, no obvious degeneration of CSMNs, striatal neurons, midbrain dopaminergic neurons, cerebellar granule cells or Purkinje cells was observed. Abnormal accumulation of acetylated α-tubulin, and autophagosome/lysosome proteins was found in the SMNs of aged cKO mice. Additionally, the total and cell surface levels of glutamate receptors were also substantially elevated in the p150^Glued^-depleted spinal neurons, in correlation with increased vulnerability to excitotoxicity.

**Conclusion:**

Overall, our findings demonstrate that p150^Glued^ is particularly required to maintain the function and survival of SMNs during aging. P150^Glued^ may exert its protective function through regulating the transportation of autophagosomes, lysosomes, and postsynaptic glutamate receptors in neurons.

**Electronic supplementary material:**

The online version of this article (10.1186/s13024-018-0242-z) contains supplementary material, which is available to authorized users.

## Background

Impairments in intracellular transport are often associated with neurological disorders, including Alzheimer’s disease, Huntington’s disease, Parkinson’s disease and amyotrophic lateral sclerosis (ALS) [1]. Dynein mediates the retrograde transport of cargos from microtubule plus ends to the minus ends, while the dynactin protein complex is proposed to promote the processivity of dynein motor proteins moving along the microtubules, as well as expand the variety of dynein cargoes [2, 3]. Dynactin consists of more than 20 subunits, with its largest subunit p150^Glued^ to interact with both microtubules and dynein motor protein complex [2]. P150^Glued^, encoded by the full-length *DCTN1* gene*,* weights around 150 kD and contains the N-terminal CAP-Gly and basic domains, followed by the coiled-coil 1 (CC1) and CC2 domains [2]. Both the CAP-Gly and basic domains exhibit microtubule binding affinity, and together form the tandem MTBDs [4]. On the other hand, the CC1 and CC2 domains mediate the interactions with dynein intermediate chain (DIC) and the other dynactin subunits [2]. The MTBDs of p150^Glued^ are required for cell division by providing essential attachment to the microtubules in spindle formation and chromosome movement [5], p150^Glued^ inhibition causes cell proliferation arrest [6] and germline deletion of p150^Glued^ leads to early embryonic lethality [7]. Multiple missense mutations in the CAP-Gly domain of p150^Glued^ have been linked to a slowly progressive, autosomal dominant form of lower motor neuron disease without sensory symptoms [8]; Perry syndrome, which consists of parkinsonism with severe mental depression and central hypoventilation [9]; and progressive supranuclear palsy [10]. P150^Glued^ is proposed as an anti-catastrophe factor in maintaining the stability of microtubules in neurons, and facilitate the dynein-mediated axonal retrograde transport in neuronal cultures [11]. These disease-causal mutations seem to weaken the microtubule binding affinity of p150^Glued^ [3]. However, the functional significance of p150^Glued^ has not been critically evaluated in neurons in living animals.

In addition to p150^Glued^, *DCTN1* also encodes p135 and other short splicing variants [12]. P135 lacks the coding exons 2 to 5, resulting in a complete loss of the CAP-Gly domain and a large portion of basic domain. P150^Glued^ is expressed in all types of mammalian cells, while p135 is more abundant in neurons [13]. Dynein/dynactin is required for the mitosis as germline deletion of p150^Glued^ caused early embryonic lethality and apoptosis in p150^Glued^ knockout mice [7]. However, p135 can compensate for the most dynactin activity in p150^glued^-deficient post-mitotic cells [12]. These observations raise questions about the overall importance of p150^Glued^ in neurons, despite mutations in its CAP-Gly domain are associated with multiple neurological disorders. One possible scenario is that p150^Glued^ proteins are particularly needed to maintain the integrity of microtubule network and the efficiency of axonal retrograde transport in the large projection neurons with long axons, such as the CSMNS and SMNs.

In this study, we utilized Cre-loxP system [14] to selectively deplete p150^Glued^ but keep p135 expression in CSMNs and SMNs. To our surprise, genetic ablation of p150^Glued^ in postnatal neurons did not cause overt behavioral and neuropathological phenotypes in mice. Only moderate motor deficits and SMN loss were observed in aged p150^Glued^ cKO mice. The p150^Glued^-lacking neurons appeared to be more susceptible to excitotoxicity in correlation with abnormal augmentations of total and surface expression of glutamate receptors, a potential pathogenic mechanism of SMN degeneration in p150^Glued^ cKO mice.

## Methods

### Generation of *Dctn1*^LoxP/^ knock-in mice and *Dctn1*^LoxP/LoxP^; *Cre* conditional knockout mice

**Fig. 1:**
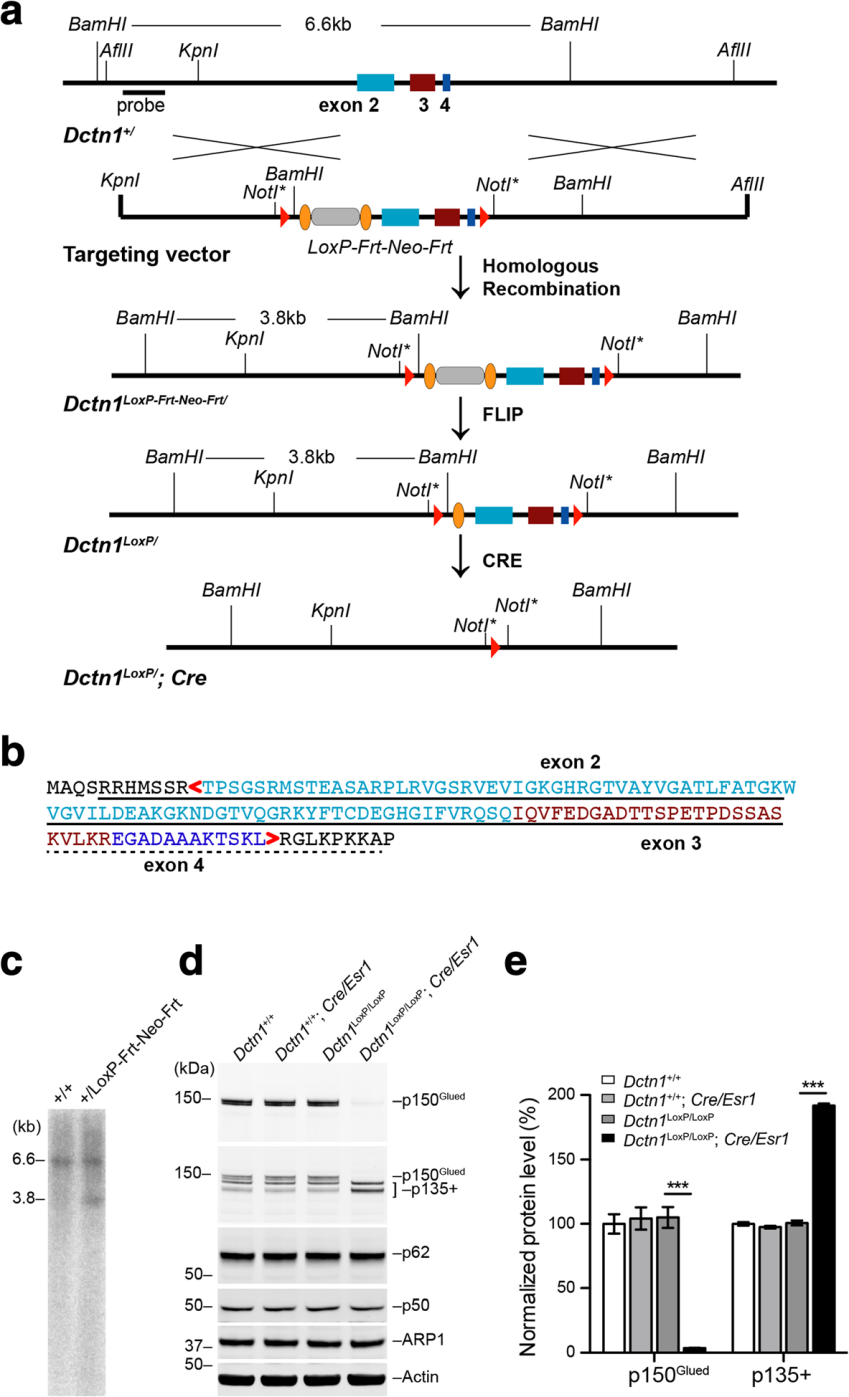
Genetic deletion of p150^Glued^ in *Dctn1*^LoxP/^; *Cre* mice. **a** The schematic diagram depicts the generation of *Dctn1*^LoxP/^; *Cre* mice in which the exon 2–4 of *Dctn1* gene is deleted. **b** Amino acid sequence highlights residues encoded by exon 2 (light blue), exon 3 (brown), and exon 4 (navy blue). The CAP-Gly domain was marked by solid line. The basic domain was indicated by dash line. **c** Southern blot of genomic DNAs isolated from mouse embryonic stem (ES) cell clones shows a correct gene targeting of the *Dctn1* gene locus in *Dctn1*^+/LoxP-Frt-Neo-Frt^ ES cell clones. As predicted, the insertion of the LoxP-Frt-Neo-Frt gene-targeting cassette into the *Dctn1* gene locus generated a new 3.8 kb *BamHI* fragment in *Dctn1*^+/LoxP-Frt-Neo-Frt^ ES cell clones. **d**-**e** Western blot analysis shows the protein levels of p150^Glued^, p135+, p62, p50 and ARP1 in cultured *Dctn1*^+/+^, *Dctn1*^+/+^; *Cre/Esr1, Dctn1*^LoxP/LoxP^ and *Dctn1*^LoxP/LoxP^; *Cre/Esr1* cortical neurons at 7 days in vitro (DIV). Actin was used as loading control. Bar graphs show the quantification of p150^Glued^ and p135+ levels normalized with actin (*n* = 3 per genotype). Data were presented as mean ± SEM. One-way ANOVA plus Tukey’s post hoc test was used for statistical analysis. ^***^p < 0.001 for difference against *Dctn1*^LoxP/LoxP^; *Cre/Esr1*, while no difference was found among *Dctn1*^+/+^, *Dctn1*^+/+^; *Cre/Esr1 and Dctn1*^LoxP/LoxP^

Genomic DNA fragments containing *Dctn1* gene locus were isolated from a mouse genomic DNA library (Stratagene). A 9.3 kb KpnI/AflII fragment carrying exons 2–4 of *Dctn1* was subcloned into the pBluescript vector for later modifications. To construct the targeting vector, the *Dctn1* clone was modified by inserting the first loxP site and a neomycin (Neo) selection cassette flanked with two Frt sites in intron 1, and the second loxP site in intron 4 (Fig. 1a). The targeting vector was linearized at a unique NotI site and transfected into 129/SvJ ES cells. After neomycin resistance (Neo) positive selection with G418 for 7 days, ES clones that had undergone homologous recombination were picked and screened by PCR, Southern blot analysis and partial genome sequencing (Fig. 1b). Two positive ES clones were expanded and injected into blastocysts. The resulting male chimera mice were bred with wild-type C57BL/6 J female mice to obtain *Dctn1*^LoxP-Frt-Neo-Frt^ mice. The *Dctn1*^LoxP-Frt-Neo-Frt^ mice were then crossbred with FLPe knock-in mice [15] (JAX, stock number: 003946) to remove Neo selection marker and get *Dctn1*^LoxP/^ mice. Finally, the *Dctn1*^LoxP/^ mice were crossed with *Thy1-Cre* [16] (JAX, Stock Number: 006143) or *Cre/Esr1* [17] (JAX, Stock Number: 004682) mice to delete exons 2 to 4 and thereby the MTBD-containing p150^Glued^ from the Cre-expressing cells. The mice were housed in a 12 h light/dark cycle and fed regular diet ad libitum. All mouse work followed the guidelines approved by the Institutional Animal Care and Use Committees of the National Institute on Aging, NIH.

### Genotyping

Genomic DNA was prepared from tail biopsy using DirectPCR Lysis Reagent (Viagen Biotech) and subjected to PCR amplification using specific sets of PCR primers for each genotype, including *Dctn1*^LoxP/^ knock-in mice (mDCTN1-Ex4-F, CAG CTG CAA AGA CCA GCA AA; mDCTN1-Ex5-R: CAC ACC ACC TTC TTA GGC TTC A), *Cre* transgenic mice (CRE-F, CAT TTG GGC CAG CTA AAC AT; CRE-R, TGC ATG ATC TCC GGT ATT GA).

### Sucrose density gradient fractionation and sedimentation analysis

Neuronal cells or neural tissues were homogenized in 50 mM Tris-HCl, pH 7.5, containing 150 mM NaC1, 1 mM EDTA and Protease Inhibitor Cocktail (Thermo Scientific). The cytosolic extracts were prepared by centrifugation at 16,000 × g for 15 min at 4 °C, and further clarified at 100,000 × g for 15 min. The resulting supernatant was layered over a 12 ml, 5–20% sucrose density gradient and centrifuged at 120,000 × g for 18 h at 4 °C. 1-ml gradient fractions were collected and equal volumes of all fractions analyzed by SDS-PAGE followed by Western blotting.

### Behavior tests

Open-field test. As described previously [18], the ambulatory and rearing activities of male mice were measured by the Flex-Field Activity System (San Diego Instruments). Flex-Field software was used to trace and quantify mouse movement in the unit as the number of beam breaks per 30 min.

Rotarod test. As described previously [19], male mice were placed onto a rotating rod with auto-acceleration from 0 rpm to 40 rpm for 1 min (San Diego Instruments). The length of time the mouse stayed on the rotating rod was recorded. Three measurements were taken for each animal during each test.

Hindlimb clasping test. In the hindlimb clasping test, male mice were suspended by the tail and observed for 10 s. The severity of hindlimb clasping phenotype was scored as follows: 0 = both hindlimbs consistently spread outward and away from the abdomen, 1 = one hindlimb retracted toward the abdomen, 2 = both hindlimbs partially retracted toward the abdomen, 3 = both hindlimbs completely retracted toward the abdomen. For each mouse, three separate tests were taken over 3 h, and the averaged hindlimb clasping severity score of the three tests was calculated.

### Histology, immunohistochemistry and light microscopy

For neuropathology study, mice were perfused with 4% paraformaldehyde (PFA) in cold phosphate buffered saline (PBS), brains and spinal cords were collected, post-fixed overnight, submerged in 30% sucrose in PBS for at least 72 h, and sectioned at 40–50 μm thickness using CM1950 cryostat (Leica). For muscle pathology analysis, mice were euthanized, gastrocnemius muscles were dissected, snap frozen in 2-methylbutane (Sigma-Aldrich) cooled in dry ice, sectioned at 20 or 40 μm thickness, and fixed in 4% PFA in PBS for 15 min. For Nissl staining, sections were stained with 0.1% Cresyl Violet (Sigma-Aldrich). For HE staining, hematoxylin and eosin stain kit (Vector Laboratories) were used as suggested by manufacturers. For immunostaining, antibodies specific to p150^Glued^ N-terminus (amino acid 3–202, 1:200, BD Biosciences), p150^Glued^ C-terminus (amino acid 1266–1278, 1:500, Abcam), neuron-specific nuclear protein (NeuN, 1:1000, Millipore), CTIP2 (1:200, Abcam), choline acetyltransferase (CHAT, 1:500, Millipore), Glial Fibrillary Acidic Protein (GFAP, 1:1000, Abcam), Ionized calcium-Binding Adaptor Molecule-1 (IBA1, 1:1000, Wako Chemicals), acetylated α-tubulin (ac-TUBA, 1:500, Abcam), Lysosomal Associated Membrane Protein 2 (LAMP2, 1:200, Abcam), Tyrosine Hydroxylase (TH, 1:500, Pel-Freeze) and Calbindin D28K (1:200, Abcam) were used as suggested by manufacturers. For immunofluorescence study, Alexa Fluor 488-, 546- or 647-conjugated secondary antibody (1:500, Invitrogen) was used to visualize the staining. Alexa Fluor 488-conjugated α-bungarotoxin (1:500, Invitrogen) was applied to label postsynaptic acetylcholine receptors of NMJs. Fluorescence images were captured using LSM 780 laser-scanning confocal microscope (Zeiss). The paired images in all the figures were collected at the same gain and offset settings. Post collection processing was applied uniformly to all paired images. The images were presented as either a single optic layer after acquisition in z-series stack scans at 1.0 μm intervals from individual fields or displayed as maximum-intensity projections to represent confocal stacks. For immunohistochemistry study, Vectastain Elite ABC Kit and DAB Kit (Vector Laboratories) were used to visualize the staining. Bright field images were captured by Axio microscope Imager A1 (Zeiss).

### Image analysis

For the quantitative assessment of various marker protein distributions, images were taken using identical settings and exported to ImageJ (NIH) for imaging analysis. Images were converted to an 8-bit color scale (fluorescence intensity from 0 to 255) using ImageJ. The areas of interest were first selected with Polygon or Freehand selection tools and then subjected to measurement by mean optical intensities or area fractions. The mean intensity for the background area was subtracted from the selected area to determine the net mean intensity.

### Stereology

Stereology for CSMNs, striatal neurons, SMNs and midbrain dopaminergic neurons was performed as described previously [19, 20]. To examine the number of CSMNs and striatal neurons, series of sagittal brain sections (40 μm per section thickness, every ninth section, nine sections per case) were processed for Nissl staining or CTIP2 immunohistochemistry. The motor cortex was designated as layer V neurons in the anteromedial cortex, and outlined according to the mouse brain in stereotaxic coordinates. To examine the number of SMNs, series of coronal spinal cord sections (50 μm per section, every tenth section from L3 to L5, ten sections per case) were processed for Nissl staining or CHAT immunohistochemistry. To examine the number of midbrain dopaminergic neurons, series of coronal sections across the midbrain (40 μm per section, every fourth section from Bregma − 2.54 to − 4.24 mm, ten sections per case) were processed for TH immunohistochemistry. For the unbiased stereological estimation of CSMNs, striatal neurons, SMNs and midbrain dopaminergic neurons, the number of CTIP2 positive or Nissl-stained large (>15 μm in diameter) cortical neurons in layer V, Nissl-stained neurons in stratum, CHAT positive or Nissl-stained large (>35 μm in diameter) spinal neurons in ventral horn, and TH-positive neurons in substantia nigra pars compacta (SNpc) were assessed using the Optical Fractionator function of Stereo Investigator 10 (MicroBrightField). Six mice were used per genotype at each time point. Counters were blinded to the genotypes of the samples. The sampling scheme was designed to have a coefficient of error less than 10% in order to obtain reliable results.

### Preparation of postsynaptic density (PSD) fraction

PSD fractions were prepared from mouse brains as described previously [18]. All procedures were performed at 4 °C. Mouse brains were isolated and homogenized in 10 volumes of ice-cold Buffer A (0.32 M sucrose, 5 mM HEPES, pH 7.4, 1 mM MgCl_2_, 0.5 mM CaCl_2_, Protease and Phosphatase Inhibitor Cocktail) with Teflon homogenizer (12 strokes). The homogenate was spun at 1400 × *g* for 10 min. Supernatant (S1’) was saved and pellet (P1’) was homogenized again with Teflon homogenizer in another 10 volumes of Buffer A (5 strokes). After centrifugation at 700 × *g* for 10 min, the supernatant (S1’) was collected and pooled with S1, and the pellet (P1’) were saved as crude nuclear fraction. Pooled S1 and S1’ were centrifuged at 13,800 × *g* for 10 min to collect the crude synaptosomal pellet (P2) and the supernatant (S2), which contains the cytosol and light membranes. S2 was centrifuged at 100,000 × g for 15 min to separate the cytosolic fraction (Cyt) and the light membrane fraction (LM). P2 was resuspended in 10 volumes of Buffer B (0.32 M sucrose, 6 mM Tris, pH 8.0, supplemented with Protease and Phosphatase Inhibitor Cocktail) with Teflon homogenizer (5 strokes). The P2 suspension was loaded onto a discontinuous sucrose gradient (0.85 M/1 M/1.15 M sucrose solution in 6 mM Tris, pH 8.0), and centrifuged at 82,500 × *g* for 2 h. Synaptosome fraction (Syn) was collected from the layer between 1 M and 1.15 M sucrose, supplemented with 0.5% Triton X-100 and mixed for 15 min. The suspension was spun at 32,800 × *g* for 20 min, and the resulting pellet was saved as PSD fraction (PSD). P1’, LM and PSD fractions were lysed in 1% SDS buffer. SDS were added into Cyt and Syn fraction to 1% final concentration. Equal amount of total protein from each fraction were resolved in SDS-PAGE and applied to western blot analysis.

### Primary neuron culture

Mouse primary neuron cultures were prepared from the cortex or spinal cord of newborn (postnatal day 0, P0) pups, as described previously [21, 22]. Briefly, individual cortex or spinal cord was dissected and subjected to papain digestion (5 U/ml, Worthington Biochemicals) for 40 min at 37 °C. The digested tissue was carefully triturated into single cells using increasingly smaller pipette tips. The cells were then centrifuged at 250 × g for 5 min and resuspended in warm Basal Medium Eagle (BME, Sigma-Aldrich) supplemented with 5% heat-inactivated fetal bovine serum (FBS; Invitrogen), 1× N2/B27 supplement (100× stock, Invitrogen), 1× GlutaMax (100× stock, Invitrogen), 0.45% D-glucose (Sigma-Aldrich), 10 U/ml penicillin (Invitrogen), and 10 μg/ml streptomycin (Invitrogen). The dissociated neurons were seeded in plated in Biocoat Poly-D-Lysine Cellware plate (BD Biosciences), and maintained at 37 °C in a 95% O_2_ and 5% CO_2_ humidified incubator. Twenty-four hours after seeding, the cultures were switched to serum-free medium supplemented with 1 μM cytosine β-D-arabinofuranoside (Sigma-Aldrich) to suppress the proliferation of glia and 1 μM 4-hydroxytamoxifen (4-OHT, Sigma-Aldrich) to induce CRE recombinase activity. Starting from 5 days in vitro (DIV), culture medium was changed twice every week.

### Biotinylation of neuronal surface proteins

Spinal neurons were cultured at the density of 1.2 × 10^6^ per well in Poly-D-Lysine coated 6-Well plate (BD Biosciences) and treated with 0 or 10 μM glutamate (Sigma-Aldrich) on 20 DIV. After 24-h treatment, neurons were washed with PBS containing 0.1 mM CaCl_2_ and 1 mM MgCl_2_ (PBS/CM), and then incubated with 1 ml biotin solution (0.5 mg/ml Sulfo-NHS-SS-Biotin in cold PBS/CM, Thermo Fisher Scientific) for 20 min at 4 °C and then washed subsequently with PBS/CM, 0.1 M glycine solution, and TBS (25 mM Tris-HCl, pH 7.4, containing 137 mM NaCl) buffer. Cells were harvested in 250 μl RIPA buffer (Sigma) supplemented with protease inhibitor and phosphatase inhibitor cocktail, lysed on ice for 30 min, and centrifuged at 16,000 × g for 15 min at 4 °C. Resulting supernatant containing equal amount of total protein was incubated with 50 μl neutral-avidin agarose (Pierce) at 4 °C for 2 h with gently rotating. After washing in RIPA buffer for 5 times, the biotin-labeled surface protein was eluted with SDS sample buffer by heating at 70 °C for 10 min. Total proteins and isolated biotinylated surface proteins were analyzed by western blotting.

### Assessment of cell survival by 3-(4, 5-Dimethylthiazol-2-yl)-2, 5-diphenyl-tetrazolium bromide (MTT) assay

Spinal neurons were cultured at the density of 1.0 × 10^5^ per well in Poly-D-Lysine coated 96-Well plate (BD Biosciences) and treated with 0 or 10 μM glutamate (Sigma-Aldrich) on 20 DIV. After 24-h treatment, the cells were incubated with 0.5 mg/ml MTT (Sigma-Aldrich) at 37 °C for 4 h. After the media were removed, dimethyl sulfoxide (DMSO, 100 μl) was added to each well to solubilize the formazan crystals generated by viable mitochondrial succinate dehydrogenase from MTT. The absorbance at 570 nm was measured using a SpectraMax M5 Multi-Mode Microplate Reader (Molecular Devices) as the MTT reducing activity of the cells.

### Western blot analysis

Neurons or tissues were homogenized and sonicated in RIPA buffer (Sigma-Aldrich) or 1% SDS lysis buffer (50 mM Tris–HCl, 150 mM NaCl, 2 mM EDTA, pH 7.5, and 1% SDS) supplemented with Protease Inhibitor Cocktails (Thermo Scientific). Lysates were clarified by centrifugation at 15000 × g for 15 min at 4 °C. The supernatants were quantified for protein content using the bicinchoninic acid (BCA) assay kit (Thermo Fisher Scientific) and separated by 4–12% NuPage BisTris-PAGE (Invitrogen) using MES or MOPS running buffer (Invitrogen). The separated proteins were then transferred to nitrocellulose membranes using the iBlot Dry Blotting system (Invitrogen) and incubated with specific primary antibodies. The antibodies used for western blot analysis included p150^Glued^ N-terminus (amino acid 3–202, 1:1000, BD Biosciences), p150^Glued^ C-terminus (amino acid 1266–1278, 1:1000, Abcam), dynactin subunit p62 (1:1000, Abcam), dynactin subunit p50 (1:5000, BD Biosciences), dynactin subunit Actin Related Protein 1 (ARP1, 1:1000, Sigma-Aldrich), dynein heavy chain (DHC, 1:500, Santa Cruz Biotechnology), dynein intermediate chain (DIC, 1:1000, Sigma-Aldrich), dynein light chain (DLC, 1:500, Santa Cruz Biotechnology), α-tubulin (TUBA, 1:10000, Abcam), acetylated α-tubulin (ac-TUBA, 1:10000, Abcam), tyrosinated α-tubulin (tyro-TUBA, 1:10000, Abcam), detyrosinated α-tubulin (detyro-TUBA, 1:10000, Abcam), Autophagy Related Protein ATG3 (ATG3, 1:1000, Cell Signaling), ATG5 (1:1000, Cell Signaling), ATG7 (1:1000, Cell Signaling), autophagy related protein LC3 (1:1000, Abcam), Lysosomal Associated Membrane Protein 1 (LAMP1, 1:1000, BD Biosciences), LAMP2 (1:1000, Abcam), synaptophysin (SYP, 1:10000, Millipore), Postsynaptic Density Protein 95 (PSD95, 1:1000, Sigma-Aldrich), AMPA receptor subunit GLUR1 (1:1000, Abcam), GLUR2 (1:1000, BD Biosciences), NMDA receptor subunit NR2A (1:250, BD Biosciences), NR2B ((1:500, BD Biosciences), and β-actin (1:5000, Sigma-Aldrich). Protein signals were visualized by IRDye secondary antibodies and Odyssey system (LI-COR Biosciences), and quantified with NIH ImageJ software.

### Statistical analysis

Statistical analysis was performed using GraphPad Prism 5 (GraphPad Software). Data were presented as mean ± SEM. Statistical significance was determined by comparing means of different groups using unpaired t-test, one-way or two-way ANOVA followed by the post hoc test. ^*^p < 0.05, ^**^p < 0.01, ^***^p < 0.001, ^****^p < 0.0001.

## Results

### Selective depletion of p150^Glued^ expression in neurons

To selectively disrupt the expression of p150^Glued^, we initially generated *Dctn1*^LoxP^ knock-in mice by inserting two *LoxP* sites in the first and fourth introns of mouse *Dctn1* gene locus, respectively, resulting in a complete loss CAP-Gly domain and partial loss of basic domain (Fig. 1a-c). The *Dctn1*^LoxP^ knock-in mice were then crossbred with *Cre/Esr1* mice to generate *Dctn1*^LoxP/LoxP^; *Cre/Esr1* pups and littermate controls for neuronal cultures. The administration of tamoxifen activated the estrogen receptor (ESR) tagged-Cre and removed the floxed exons, resulting in a selective ablation of p150^Glued^ expression in cortical neurons cultured from postnatal day 0 (P0) *Dctn1*^LoxP/LoxP^; *Cre/Esr1* pups (Fig. 1d, e). P150^Glued^ proteins were identified by an antibody against the N-terminal 200 amino acids of p150^Glued^. By contrast, the levels of p135 and other N-terminal truncated p150^Glued^, called collectively as p135+, were substantially increased (Fig. 1d, e). The p135+ proteins were recognized by an antibody against the C-terminus of p150^Glued^. On the other hand, the other dynactin subunits–p62, p50, and ARP1 showed comparable expression levels in *Dctn1*^LoxP/LoxP^; *Cre/Esr1* and control neuronal cultures (Fig. 1d, e). Therefore, genetic depletion of p150^Glued^ increases p135+ expression, but does not affect the other dynactin subunit levels in neurons.

### A lack of p150^Glued^ does not affect the formation of dynein and dynactin complexes

**Fig. 2:**
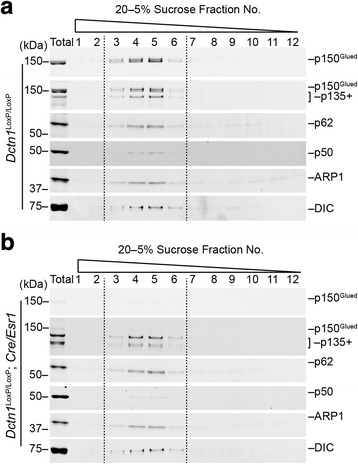
Dynein/dynactin complex remains intact in *Dctn1*^LoxP/LoxP^; *Cre/Esr1 neurons*. **a**-**b** Sucrose density gradient centrifugation shows p150^Glued^ and p135+ predominantly migrate at fraction 4 and 5 in a 5–20% sucrose gradient similar to the other dynactin subunits p62, p50, and ARP1, as well as the dynactin-interacting dynein subunit DIC in the control *Dctn1*^LoxP/LoxP^ (**a**) and p150^Glued^ cKO *Dctn1*^LoxP/LoxP^; *Cre/Esr1* cortical neuron cultures (**b**) at 7 DIV

To evaluate the impact of p150^Glued^ on the formation of dynein/dynactin complexes, we fractionated the cell lysates from *Dctn1*^LoxP/LoxP^; *Cre/Esr1* and control *Dctn1*^LoxP/LoxP^ cortical neuron cultures in a sucrose gradient column and compared the distribution patterns of dynein and dynactin subunits in different fractions. In control cell lysates, all the dynactin subunits and dynein DIC subunit concentrated in fractions 3 to 6 (Fig. 2a). The same distribution pattern of dynein and dynactin subunits was also observed in the p150^Glued^-lacking cell lysates (Fig. 2b). Thus, a lack of p150^Glued^ does not affect the formation of dynein/dynactin complexes.

### Generation of p150^Glued^ conditional knockout mice that lack p150^Glued^ expression in the postnatal forebrain and spinal neurons

Our previous study suggests the G59S missense mutation in the MTBDs of p150^Glued^ might cause the autosomal dominant motor neuron disease through a dominant-negative mechanism [7]. On the one hand, the G59S substitution disrupts the normal folding and destabilizes the mutant p150^Glued^ proteins. On the other hand, the residual mutant proteins might dimerize with the wild-type proteins and thereby compromises the overall function of p150^Glued^. However, the exact pathogenic mechanisms of G59S mutation remain controversial [1]. To test the hypothesis that p150^Glued^ is required for the survival of motor neurons, we decided to remove p150^Glued^ from both CSMNs in the frontal cortex and SMNs in the ventral spinal cords by generating *Dctn1*^LoxP/LoxP^; *Thy1-Cre* cKO mice. In line with the early study [16], Cre was widely expressed by neurons in the forebrain regions (Additional file 1: Figure S1A), including olfactory bulb, frontal cortex, striatum, and hippocampus, as well as in the spinal cord (Additional file 1: Figure S1B). Although the expression of Cre was detectable in the brain extracts of P0 pups, a substantial reduction of p150^Glued^ expression became apparent in 2-month-old *Dctn1*^LoxP/LoxP^; *Thy1-Cre* mice (Additional file 1: Figure S1C). The depletion of p150^Glued^ restrictive to adult neurons may avoid any potential developmental defects in the p150^Glued^ cKO mice.

**Fig. 3:**
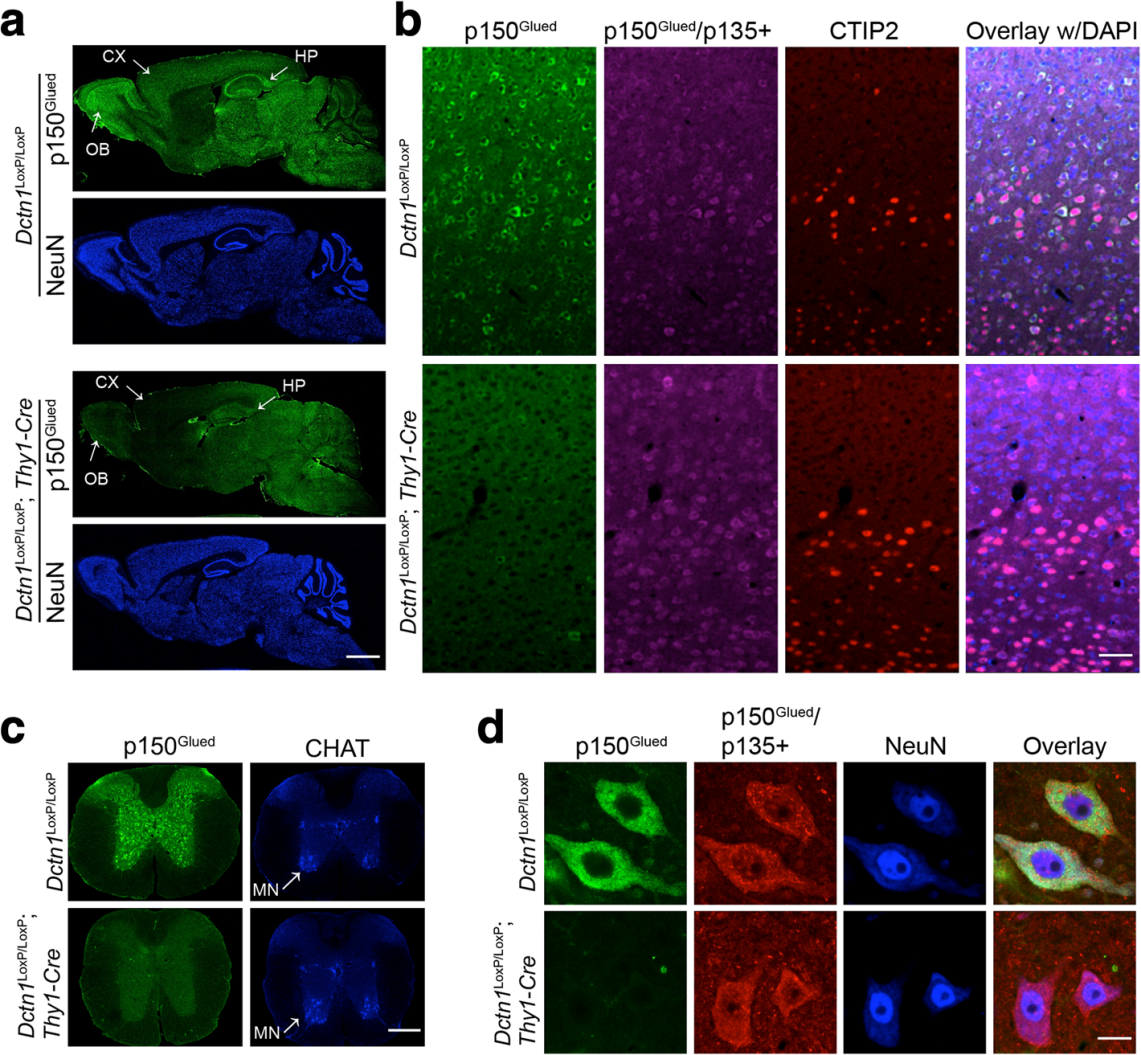
Selective deletion of neuronal p150^Glued^ in brain and spinal cord of *Dctn1*^LoxP/LoxP^; *Thy1-Cre* mice. **a** Representative images show p150^Glued^ (green) and NeuN (blue) staining in the sagittal brain sections of 3-month-old *Dctn1*^LoxP/LoxP^ and *Dctn1*^LoxP/LoxP^; *Thy1-Cre* mice. Arrows point to olfactory bulb (OB), cortex (CX) and hippocampus (HP) with intense p150^Glued^ staining in *Dctn1*^LoxP/LoxP^ mice and weak p150^Glued^ staining in *Dctn1*^LoxP/LoxP^; *Thy1-Cre* mice. Scale bar: 500 μm. **b** Representative images show p150^Glued^ (green) and p150^Glued^/p135+ (purple) expression in the CTIP2-positive (red) CSMNs of 3-month-old *Dctn1*^LoxP/LoxP^ and *Dctn1*^LoxP/LoxP^; *Thy1-Cre* mice. Scale bar: 500 μm. **c** Representative images show p150^Glued^ (green) and CHAT (blue) staining in the lumbar spinal cord sections of 3-month-old *Dctn1*^LoxP/LoxP^ and *Dctn1*^LoxP/LoxP^; *Thy1-Cre* mice. Arrows point to the CHAT-positive SMNs. Scale bar: 500 μm. **d** Representative images show p150^Glued^ (green) and p150^Glued^/p135+ (red) expression in the NeuN-positive SMNs of 3-month-old *Dctn1*^LoxP/LoxP^ and *Dctn1*^LoxP/LoxP^; *Thy1-Cre* mice. Scale bar: 20 μm

Immuno-staining showed endogenous p150^Glued^ proteins were highly expressed by neurons in the olfactory bulb, cerebral cortex, hippocampus, midbrain, and hindbrain regions of control *Dctn1*^LoxP/LoxP^ mice (Fig. 3a). Following the expression pattern of Cre (Additional file 1: Figure S1A), the levels of p150^Glued^ were substantially depleted in the olfactory bulb, frontal cortex, and hippocampus of *Dctn1*^LoxP/LoxP^; *Thy1-Cre* cKO mice (Fig. 3a). More importantly, p150^Glued^ was absent in the CSMNs of cKO mouse brains, while the p135+ remained (Fig. 3b). Here the CSMNs were indicated by staining with CSMN-specific marker protein CTIP2 [23]. P150^Glued^ was also abundantly expressed by spinal neurons, including the SMNs in the ventral horns, but disappeared in the cKO spinal cords (Fig. 3c, d). In contrast, the p135+ proteins were still present in the SMNs of cKO mice (Fig. 3d). Therefore, we generated a line of p150^Glued^ cKO mice that depleted the expression of p150^Glued^ in the adult CSMNs and SMNs, while kept p135 and other MTBD-lacking variants.

### P150^Glued^ conditional knockout mice develop late-onset motor impairments

**Fig. 4:**
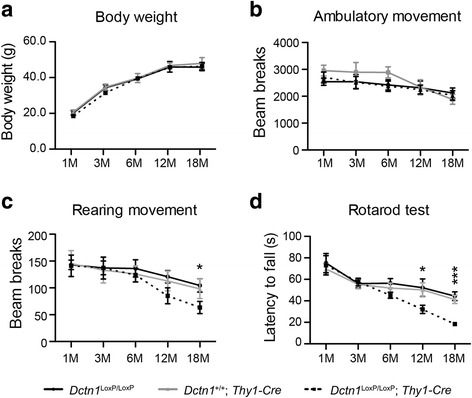
*Dctn1*^*LoxP/LoxP*^*; Thy1-Cre* mice develop abnormal motor phenotypes. **a**-**d** Male *Dctn1*^LoxP/LoxP^, *Dctn1*^+/+^*; Thy1-Cre* and *Dctn1*^LoxP/LoxP^; *Thy1-Cre* mice (*n* ≥ 10 per genotype per time point) were measured for body weight (**a**); tested for the ambulatory movement (**b**) and rearing movement (**c**) in Open-field; and examined for the latency to fall in Rotarod test (**d**) at 1, 3, 6, 12 and 18 months of age. Data were presented as mean ± SEM. One-way ANOVA plus Tukey’s post hoc test was used for statistical analysis. **c** At 18 months of age, ^*^p < 0.05 for difference against *Dctn1*^LoxP/LoxP^; *Thy1-Cre*, while no difference was found between *Dctn1*^LoxP/LoxP^ and *Dctn1*^+/+^; *Thy1-Cre.* **d** At 12 or 18 months of age, ^*^p < 0.05 or ^***^p < 0.001 for difference against *Dctn1*^LoxP/LoxP^; *Thy1-Cre*, respectively, while no difference was found between *Dctn1*^LoxP/LoxP^ and *Dctn1*^+/+^; *Thy1-Cre*

The *Dctn1*^LoxP/LoxP^; *Thy1-Cre* p150^Glued^ cKO mice were born at a normal Mendelian ratio, developed normally, and had similar body weights as littermate controls (Fig. 4a). The cKO mice also displayed normal ambulatory movement in the open-field tests (Fig. 4b), but showed markedly reduced rearing at 18 months of age (Fig. 4c), and stayed less time on the rotating rods compared to the controls starting at 12 months of age (Fig. 4d). These data suggest that a lack of p150^Glued^ affects the motor control of aged animals.

### P150^Glued^ conditional knockout mice develop late-onset, selective degeneration of SMNs

**Fig. 5:**
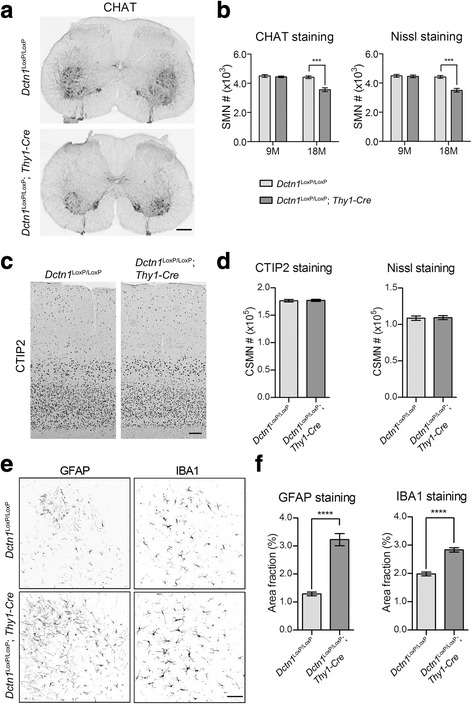
*Dctn1*^LoxP/LoxP^; *Thy1-Cre* mice display loss of spinal motor neurons and gliosis. **a** Representative images show CHAT staining in the lumbar spinal cord of *Dctn1*^LoxP/LoxP^ and *Dctn1*^LoxP/LoxP^; *Thy1-Cre* mice at 18 months of age. Scale bar: 500 μm. **b** Unbiased stereological estimation of the number of CHAT-positive SMNs and Nissl-stained SMNs in the lumbar spinal cord of 9 and 18-month-old *Dctn1*^LoxP/LoxP^ and *Dctn1*^LoxP/LoxP^; *Thy1-Cre* mice (*n* = 6 per genotype per time point). Data were presented as mean ± SEM. Two-way ANOVA plus Bonferroni’s post hoc test was used for statistical analysis. At 18 months of age, ^***^p < 0.001 for difference between *Dctn1*^LoxP/LoxP^ and *Dctn1*^LoxP/LoxP^; *Thy1-Cre*. There were significant main effects for age (CHAT staining: *F*_(1, 20)_ = 25.51, *p* < 0.0001; Nissl staining: *F*_(1, 20)_ = 25.18, *p* < 0.0001), genotype (CHAT staining: *F*_(1, 20)_ = 22.46, *p* = 0.0001; Nissl staining: *F*_(1, 20)_ = 21.85, *p* = 0.0001) and age-genotype interaction (CHAT staining: *F*_(1, 20)_ = 17.08, *p* = 0.0005; Nissl staining: *F*_(1, 20)_ = 18.47, *p* = 0.0004). **c** Representative images show CTIP2 staining in the motor cortex of *Dctn1*^LoxP/LoxP^ and *Dctn1*^LoxP/LoxP^; *Thy1-Cre* mice at 18 months of age. Scale bar: 500 μm. **d** Unbiased stereological estimation of the number of CTIP2-positive and Nissl-stained large pyramidal neurons in the layer V motor cortex of 18-month-old *Dctn1*^LoxP/LoxP^ and *Dctn1*^LoxP/LoxP^; *Thy1-Cre* mice (*n* = 6 per genotype per time point). Data were presented as mean ± SEM. Unpaired t-test showed no statistical significance (*p* > 0.05). **e** Representative images show GFAP and IBA1 staining in the ventral horn of lumbar spinal cord of *Dctn1*^LoxP/LoxP^ and *Dctn1*^LoxP/LoxP^; *Thy1-Cre* mice at 18 months of age. Scale bar: 500 μm. **f** The areas occupied by GFAP-positive astrocytes and IBA1-positive microglia in the ventral horn of lumbar spinal cord of *Dctn1*^LoxP/LoxP^ and *Dctn1*^LoxP/LoxP^; *Thy1-Cre* mice at 18 months of age (n = 6 animals and 10 sections per animal). Data were presented as mean ± SEM; ^****^p < 0.0001. Unpaired t-test was used for statistical analysis

To investigate whether p150^Glued^ depletion causes motor neuron loss, we used the unbiased stereology approach to count the numbers of lumbar SMNs and CSMNs in 9 and 18-month-old control *Dctn1*^LoxP/LoxP^ mice and *Dctn1*^LoxP/LoxP^; *Thy1-Cre* cKO mice. Series of evenly sampled lumbar spinal sections were stained with SMN marker CHAT or histological dye Nissl for neuron counting (Fig. 5a). There was modest but statistically significant reduction of CHAT-positive SMN numbers in the lumbar spinal cord of 18-month-old cKO mice (Fig. 5b). The decrease of lumbar SMN numbers in 18-month-old cKO mice was also observed in the Nissl-stained sections (Fig. 5b). On the other hand, no significant alterations of lumbar SMN numbers were found in 9-month-old cKO mice (Fig. 5b). Additionally, no apparent changes of CTIP2-positive or Nissl-stained CSMN numbers were detected in the motor cortex of 18-month-old cKO mice (Fig. 5c, d). Given that endogenous p150^Glued^ are ubiquitously expressed throughout central neuronal system, we investigated whether p150^Glued^ depletion led to neurodegeneration in other motor function-related brain regions, including midbrain, striatum and cerebellum. Unbiased stereology revealed that no obvious loss of TH-positive dopaminergic (DA) neurons in substantia nigra pars compacta (SNpc) (Additional file 1: Figure S2A, B) and striatal neurons in 18-month-old cKO mice (Additional file 1: Figure S2C-E). Furthermore, neither the thickness of molecular layer (ML) and granule cell layer (GCL), or the density of Calbindin D28K-positive Purkinje cells in the cerebellum of 18-month-old cKO mice was obviously altered (Additional file 1: Figure S2C, F-I). These results demonstrate that p150^Glued^ depletion in adult neurons causes preferentially SMN loss in aged animals.

### P150^Glued^ depletion induces astrogliosis and microgliosis in the spinal cord of aged p150^Glued^ cKO mice

Given that astrogliosis and microgliosis are often in accompany with motor neurodegeneration in the spinal cord [24], we stained the lumbar spinal sections with antibodies against astrogliosis marker GFAP and microgliosis marker IBA1. In line with previous findings [24], we observed a substantial induction of astrogliosis and microgliosis in the spinal cord of 18-month-old cKO mice (Fig. 5e, f).

### P150^Glued^ ablation causes severe neuromuscular defects in aged p150^Glued^ cKO mice

**Fig. 6:**
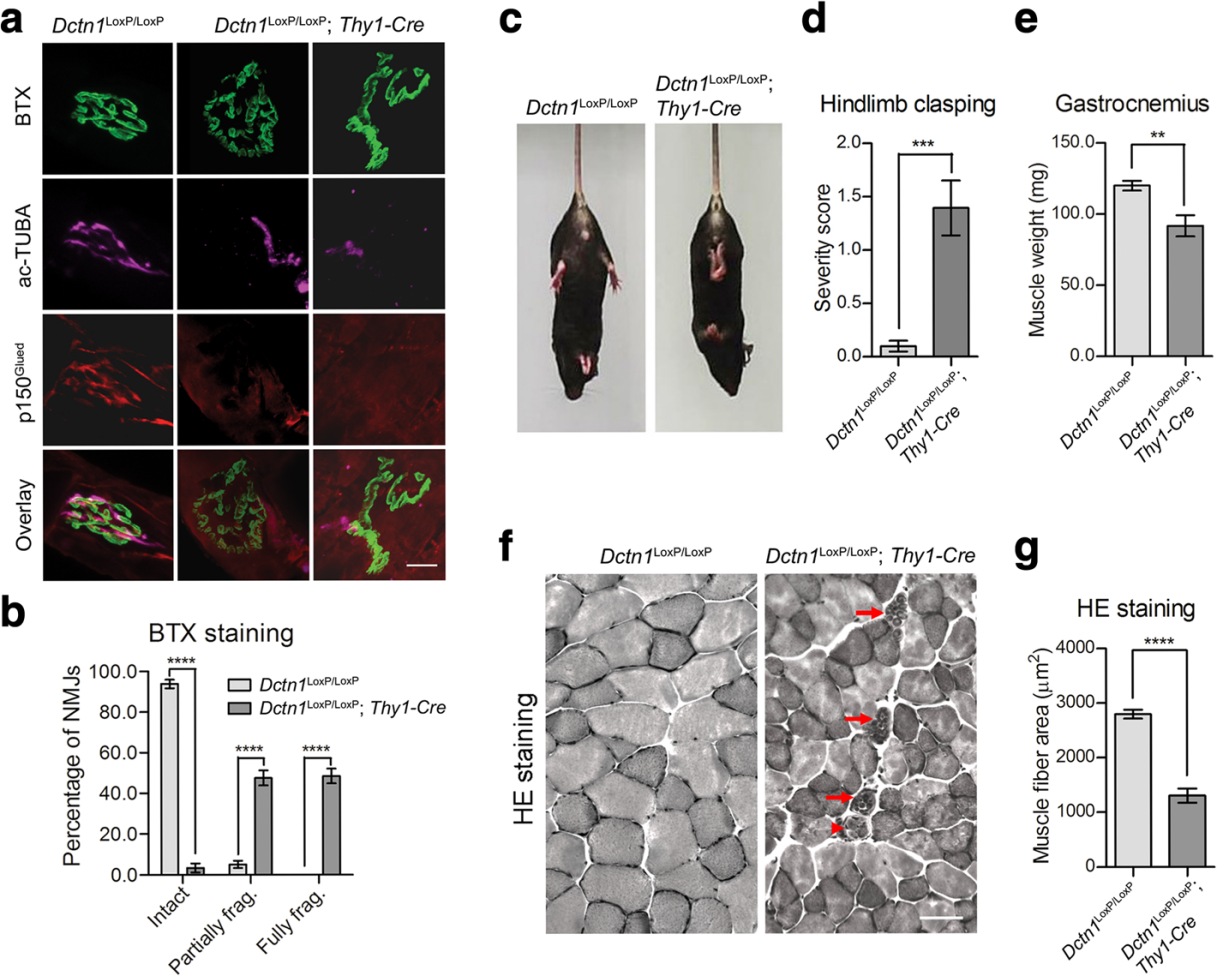
*Dctn1*^LoxP/LoxP^; *Thy1-Cre* mice show NMJ fragmentation. **a** Representative images show α-bungarotoxin (BTX, green), p150^Glued^ (red) and ac-TUBA (purple) staining in the gastrocnemius muscles of *Dctn1*^LoxP/LoxP^ and *Dctn1*^LoxP/LoxP^; *Thy1-Cre* mice at 18 months of age. Scale bar: 20 μm. **b** Bar graph shows the percentage of intact, partially fragmented, and fully fragmented NMJs in the gastrocnemius muscles of *Dctn1*^LoxP/LoxP^ and *Dctn1*^LoxP/LoxP^; *Thy1-Cre* mice at 18 months of age (n = 6 animals and 10 sections per animal). Data were presented as mean ± SEM; ^****^p < 0.0001. Unpaired t-test was used for statistical analysis. **c** Photographs display posture of 18-month-old male *Dctn1*^LoxP/LoxP^ and *Dctn1*^LoxP/LoxP^; *Thy1-Cre* mice in the hindlimb clasping test. **d** Bar graph shows the hindlimb clasping severity score of 18-month-old male *Dctn1*^LoxP/LoxP^ and *Dctn1*^LoxP/LoxP^; *Thy1-Cre* mice (*n* = 10–11 per genotype). Data were presented as mean ± SEM; ^***^p < 0.001. Unpaired t-test was used for statistical analysis. **e** Quantification of gastrocnemius muscle weight from 24-month-old male *Dctn1*^LoxP/LoxP^ and *Dctn1*^LoxP/LoxP^; *Thy1-Cre* mice (n = 10–11 per genotype). Data were presented as mean ± SEM; ^**^p < 0.01. Unpaired t-test was used for statistical analysis. **f** Representative images show hematoxylin and eosin (HE) staining of gastrocnemius cross sections from 24-month-old male *Dctn1*^LoxP/LoxP^ and *Dctn1*^LoxP/LoxP^; *Thy1-Cre* mice. Note the very high amount of small muscle fibers as well as myofiber de−/regeneration (arrow head) and inflammatory cells infiltration (arrow) in *Dctn1*^LoxP/LoxP^; *Thy1-Cre* mice. Scale bar: 50 μm. **g** Quantification of gastrocnemius muscle fiber areas from 24-month-old male *Dctn1*^LoxP/LoxP^ and *Dctn1*^LoxP/LoxP^; *Thy1-Cre* mice (n = 6 mice per genotype and 6 sections per mouse). Data were presented as mean ± SEM; ^****^p < 0.0001. Unpaired t-test was used for statistical analysis

To investigate whether p150^glued^ depletion impairs the connections between SMN axon terminals and the innervated muscles, we examined the neuromuscular junctions (NMJs) of gastrocnemius muscles of 18-month-old control and p150^Glued^ cKO mice. α-Bungarotoxin (BTX), which specifically labels acetylcholine receptors at the postsynaptic sites, was applied to label the NMJs (Fig. 6a). Meanwhile, acetylated α-tubulin (ac-TUBA), which is enriched in axons, was used to mark the axon terminals (Fig. 6a). While the NMJs in control mice remained mostly intact, the majority of NMJs in p150^Glued^ cKO mice were either partially or fully fragmented, in company with severe denervation (Fig. 6a, b). The hindlimb clasping test has been used to rate the extent of neuromuscular function impairment in rodent models of motor neuron diseases [25]. 18-month-old male control and p150^Glued^ cKO mice were suspended by the tail for 10 s and the severity of hindlimb clasping was scored. Control mice splayed their hindlimbs outward and away from the abdomen with no clasping reflex, while p150^Glued^ cKO mice retracted their hindlimbs toward the abdomen with substantially higher hindlimb clasping severity score than control mice (Fig. 6c, d). Given that denervation often induce skeletal muscle atrophy in motor neuron diseases, we measured muscle weight and examined gross muscle pathology of control and p150^Glued^ cKO mice. We observed a substantial decrease of gastrocnemius muscle weight in 24-month-old male p150^Glued^ cKO mice (Fig. 6e). Hematoxylin and eosin (HE) staining of gastrocnemius cross sections revealed that 24-month-old male control mice had a polygonal shaped muscle fibers with homogenous fiber size, whereas p150^Glued^ cKO mice exhibited significantly smaller size of muscle fiber in accompany with pathological hallmarks of myofiber de−/regenerating and inflammatory cells infiltration (Fig. 6f, g). These results demonstrate that a loss of p150^glued^ caused substantial NMJ disruption and muscle atrophy in aged mice.

### A loss of p150^Glued^ increases α-tubulin acetylation

**Fig. 7:**
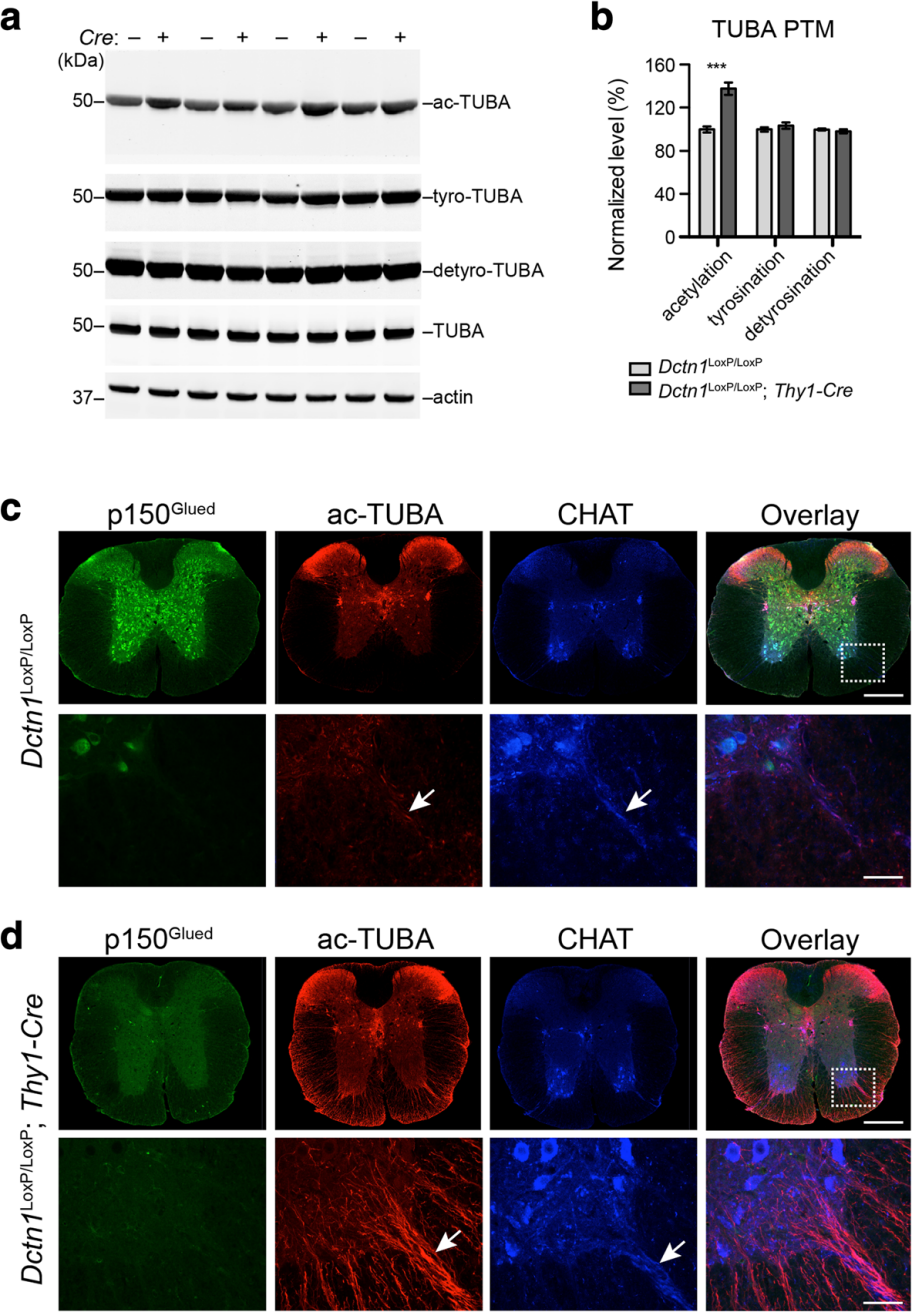
P150^Glued^ deficiency enhances tubulin acetylation in spinal motor neurons. **a**-**b** Western blot analysis shows the expression of acetylated (ac-), tyrosinated (tyro-), detyrosinated (detyro-) and total alpha tubulin (TUBA) in the spinal cord homogenate of 9-month-old *Dctn1*^LoxP/LoxP^ and *Dctn1*^LoxP/LoxP^; *Thy1-Cre* mice. Actin was used as loading control. Bar graphs show the quantification of ac-TUBA, tyro-TUBA and detyro-TUBA level normalized with total TUBA (*n* = 8 per genotype). Data were presented as mean ± SEM, ^***^p < 0.001. Unpaired t-test was used for statistical analysis. **c**, **d** Representative images show p150^Glued^ (green), ac-TUBA (red) and CHAT (blue) staining in the lumbar spinal cord of 9-month-old *Dctn1*^LoxP/LoxP^ (**c**) and *Dctn1*^LoxP/LoxP^; *Thy1-Cre* (**d**) mice. Four mice per genotype and 10 sections per mouse were examined. Arrows point to CHAT-positive ventral roots with weak ac-TUBA staining in *Dctn1*^LoxP/LoxP^ mice and substantial ac-TUBA staining in *Dctn1*^LoxP/LoxP^; *Thy1-Cre* mice. Scale bars: 500 μm (low-magnification images), 20 μm (high-magnification images)

Microtubules are formed by the polymerization of α- and β-tubulin dimers [26]. Various post-translational modifications (PTMs) of α-tubulin affect the microtubule dynamics [27]. To assess the impact of p150^Glued^ depletion on α-tubulin PTMs, we examined the levels of α-tubulin acetylation, tyrosination, and detyrosination by western blots in the spinal cord homogenates of 9-month-old *Dctn1*^LoxP/LoxP^; *Thy1-Cre* cKO mice and littermate *Dctn1*^LoxP/LoxP^ mice (Fig. 7a). We observed a substantial increase of acetylated α-tubulin levels in the p150^Glued^ cKO samples (Fig. 7a, b). We further confirm this finding by immuno-staining the lumbar spinal sections of 9-month-old mice. There were a few spots in the ventral root SMN axon bundles positive to acetylated α-tubulin staining in the control spinal cords (Fig. 7c). By contrast, a drastic upregulation of acetylated α-tubulin was found in the SMN axons of cKO mice (Fig. 7d). These data demonstrate that p150^Glued^ depletion leads to substantial increase of acetylated α-tubulin levels in the SMN axons, suggesting potential alterations of microtubule stability in p150^Glued^-lacking SMNs.

### A loss of p150^Glued^ promotes autophagosome and lysosome protein accumulation

**Fig. 8:**
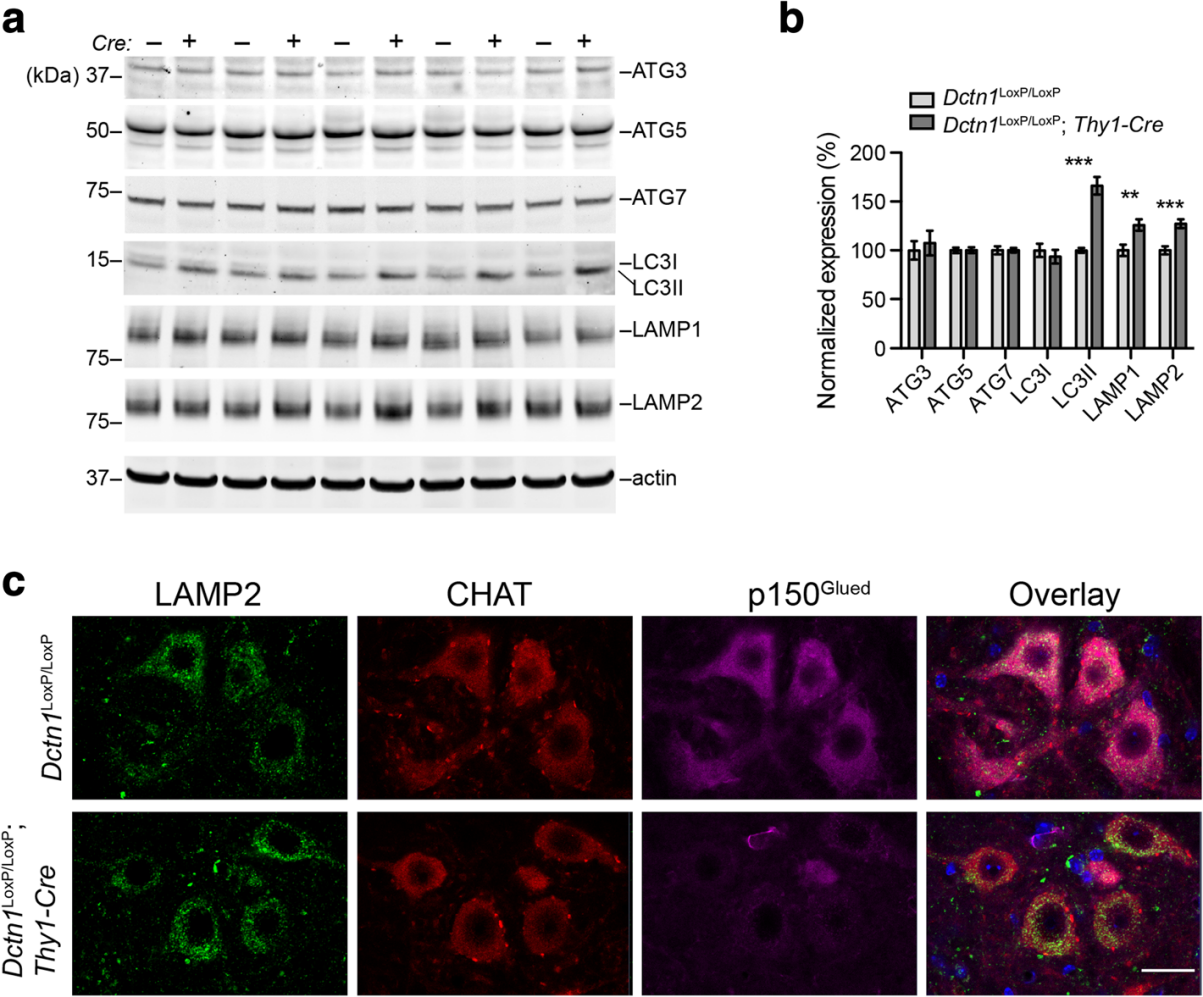
P150^Glued^ deficiency alters autophagosome and lysosome marker protein expression in spinal motor neurons. **a** Western blot analysis shows the expression of ATG3, ATG5, ATG7, LC3, LAMP1 and LAMP2 in the spinal cord homogenate of 9-month-old *Dctn1*^LoxP/LoxP^ and *Dctn1*^LoxP/LoxP^; *Thy1-Cre* mice. Actin was used as loading control. **b** Bar graphs show the quantification of ATG3, ATG5, ATG7, LC3, LAMP1 and LAMP2 level normalized with actin (*n* = 10 per genotype). Data were presented as mean ± SEM; ^**^p < 0.01, ^***^p < 0.001. Unpaired t-test was used for statistical analysis. **c** Representative images show LAMP2 (green), CHAT (red) and p150^Glued^ (purple) staining in the lumbar spinal cord of 9-month-old *Dctn1*^LoxP/LoxP^ and *Dctn1*^LoxP/LoxP^; *Thy1-Cre* mice. Four mice per genotype and 10 sections per mouse were examined. Scale bar: 20 μm

Dynein/dynactin complexes mediate the transport of autophagosomes and lysosomes in cells [28]. Early studies show impairments of autophagosome and lysosome trafficking in the SMNs of transgenic mice overexpressing disease-related p150^Glued^ G59S mutation, resulting in cytoplasmic accumulation of autophagosome and lysosome-enriched proteins [29, 30]. Accordingly, we examined the levels of autophagosome and lysosome marker proteins in the spinal cord lysates of 9-month-old p150^Glued^ control and cKO mice by western blots. Substantial increases of autophagosome protein LC3II [31] and lysosome proteins LAMP1 and LAMP2 [32] were found in the cKO spinal cords (Fig. 8a, b). However, no apparent alterations of other autophagosome proteins ATG3, ATG5, ATG7, and LC3I were detected (Fig. 8a, b). Since the LAMP2 antibody also works well in immunostaining of brain tissues, we further checked the distribution pattern of LAMP2-postive signals in the SMNs of 9-month-old control and cKO mice. We observed markedly increased LAMP2 signals in the perinuclear regions of cKO SMNs (Fig. 8c). Together, our results reveal the impairments of autophagosome and lysosome transportation in the p150^Glued^-depleted SMNs.

### P150^Glued^ and other dynactin subunits are more enriched in the postsynaptic sites compared to dynein motor proteins

**Fig. 9:**
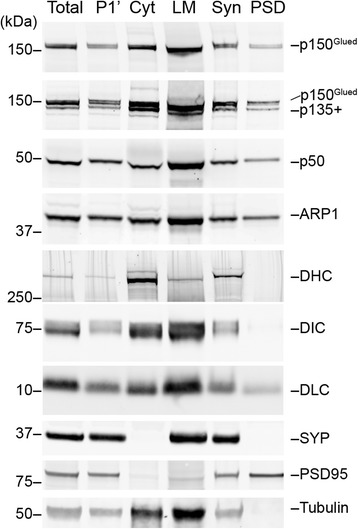
P150^Glued^ and other dynactin subunits are present in the postsynaptic sites. Subcellular fractionation of 3-month-old wild-type mouse brains shows the distribution of dyanctin subunits p150^Glued^, p135+, p50 and ARP1, dynein subunits DHC, DIC and DLC, synaptophysin (SYP), postsynaptic density protein 95 (PSD95), and α-tubulin in total brain lysates (Total), crude nuclear fraction (P1’), cytosol (Cyt), light membrane fraction (LM), synaptosomes (Syn), and postsynaptic density (PSD)

Neurons communicate with each other through synapses, where the transmitters released from the presynaptic axon terminals bind to the receptors located in the opposite postsynaptic sites in dendritic spines, small protrusions from dendritic shaft [33]. P150^Glued^ is enriched in the axon terminals and facilitate the loading of cargos to dynein-mediated axonal retrograde transport in developing neurons [34]. To investigate whether p150^Glued^ plays any role in the postsynaptic sites, we purified postsynaptic density (PSD)-enriched fractions from control mouse brains. The purity of extracted PSD fractions was verified by the presence of high levels of PSD95 proteins, but not the presynaptic protein synaptophysin (Fig. 9). Interestingly, p150^Glued^, p135, and other dynactin subunits were found in the PSD fractions, whereas dynein heavy chain (DHC), DIC, and tubulins were undetectable (Fig. 9). On the other hand, low levels of dynein light chain (DLC) were present in the PSD fraction (Fig. 9). These data suggest that p150^Glued^ and dynactin may mediate the transport of postsynaptic cargos from the postsynaptic sites to dynein motor complex associated with microtubules in the dendrites.

### P150^Glued^ depletion leads to increased cell surface targeting of glutamate receptors and increased vulnerability to glutamate-induced excitotoxicity

**Fig. 10:**
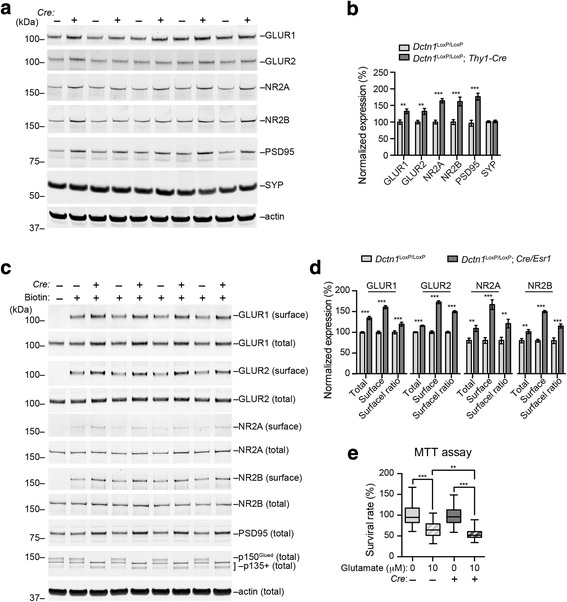
P150^Glued^ deficiency increases protein expression of glutamate receptors and vulnerability to glutamate-mediated excitotoxicity in spinal neurons. **a** Western blot analysis shows the expression of AMPAR subunits GLUR1 and GLUR2, NMDAR subunits NR2A and NR2B, postsynaptic density protein 95 (PSD95) and synaptophysin (SYP) in the spinal cord homogenate of 9-month-old *Dctn1*^LoxP/LoxP^ and *Dctn1*^LoxP/LoxP^; *Thy1-Cre* mice. Actin was used as loading control. **b** Bar graphs show the quantification of GLUR1, GLUR2, NR2A, NR2B, PSD95 and SYP level normalized with actin (n = 10 per genotype). Data were presented as mean ± SEM; ^**^p < 0.01, ^***^p < 0.001. Unpaired t-test was used for statistical analysis. **c** Western blot analysis shows the levels of biotinylated cell surface and total AMPAR subunits GLUR1 and GLUR2, NMDAR subunits NR2A and NR2B in cultured *Dctn1*^LoxP/LoxP^ and *Dctn1*^LoxP/LoxP^; *Cre/Esr1* spinal neurons at 21 DIV. Actin was used as loading control. **d** Bar graphs show the quantification of total and surface GLUR1, GLUR2, NR2A and NR2B level normalized with actin, and the ratio of GLUR1, GLUR2, NR2A and NR2B at cell surface versus total proteins (n = 8 per genotype). Data were presented as mean ± SEM; ^**^p < 0.01, ^***^p < 0.001. Unpaired t-test was used for statistical analysis. (E) Primary spinal neurons were treated with glutamate, and the viability of these neurons was measured by the MTT assay. Box & whiskers graphs show survival rate (percentage) of cultured *Dctn1*^LoxP/LoxP^ and *Dctn1*^LoxP/LoxP^; *Cre/Esr1* spinal neurons (21 DIV) treated with 0 or 10 μM glutamate for 24 h (*N* = 40 per genotype per condition). Data were presented as mean ± SEM. One-way ANOVA plus Tukey’s post hoc test was used for statistical analysis. ^***^p < 0.001 for difference between *Dctn1*^LoxP/LoxP^ neurons treated with 0 and 10 μM glutamate, ^***^p < 0.001 for difference between *Dctn1*^LoxP/LoxP^; *Cre/Esr1* neurons treated with 0 and 10 μM glutamate, ^**^p < 0.01 for difference between *Dctn1*^LoxP/LoxP^ and *Dctn1*^LoxP/LoxP^; *Cre/Esr1* neurons treated with 10 μM glutamate, while no difference was found between *Dctn1*^LoxP/LoxP^ and *Dctn1*^LoxP/LoxP^; *Cre/Esr1* neurons treated with 0 μM glutamate

Excitotoxicity mediated by glutamate and its receptors has been implicated in the pathogenesis of motor neuron diseases [35]. Using western blots, we found the levels of glutamate receptors GLUR1, GLUR2, NR2A, and NR2B, as well as postsynaptic density protein 95 (PSD95) were all substantially elevated in the spinal cord lysates of 9-month-old *Dctn1*^LoxP/LoxP^; *Thy1-Cre* cKO mice (Fig. 10a, b). By contrast, we did not observe any obvious changes of presynaptic protein synaptophysin expression (Fig. 10a, b). To investigate whether more glutamate receptors are transported to the synapses, we isolated the biotin-labeled cell surface proteins from cultured spinal neurons, and found substantial elevation of glutamate receptor GLUR1, GLUR2, NR2A and NR2B present at the membrane fractions (Fig. 10c, d). In correlation with the increased cell surface targeting of glutamate receptors, p150^Glued^ cKO neurons showed more severe cell death after treated with 10 μm glutamate (Fig. 10e). Therefore, p150^Glued^ depletion causes more postsynaptic accumulation of glutamate receptors, making p150^Glued^-lacking neurons more susceptible to excitotoxicity.

## Discussion

In the current study, we generated and characterized a line of p150^Glued^ cKO mice to critically evaluate the roles of CAP-Gly domain-containing dynactin p150^Glued^ in postnatal neurons. A loss of p150^Glued^ in postnatal neurons did not affect the assembly of dynein/dynactin motor protein complex in neurons, but caused rather selective SMN degeneration and movement impairments in aged mice. The depletion of p150^Glued^ also led to substantial elevation of acetylated α-tubulin, glutamate receptors, and proteins involved in autophagosome and lysosome pathways in the cKO neurons. Finally, p150^Glued^ ablation rendered spinal neurons more susceptible to excitotoxicity. Considering all the disease-causative mutations compromise the MTBD functions of p150^Glued^, increased excitotoxicity may underlie a common pathogenic mechanism of p150^Glued^ mutations in diverse neurodegenerative diseases.

The absence of more drastic morphological and functional phenotypes in p150^Glued^ cKO neurons suggests that p135 and other MTBD-lacking p150^Glued^ splicing variants may largely substitute the functions of p150^Glued^ in neurons. The CC1 domain of p150^Glued^ mediates the interaction with DIC and other dynein motor protein complex [2]. Since p135 and other p150^Glued^ short forms contain the CC1 domain, it is not surprising that dynein and dynactin complex remains intact and appears mostly functional in neurons. P150^Glued^ thereby is likely devoted to some special tasks in neurons. Neurons, especially the large projection neurons like CSMNs and SMNs, contain long axons. Microtubules form the backbone of these axons. P150^Glued^ is proposed to play a critical role in maintaining the stability of microtubules by serving as an anti-catastrophe factor in neuronal cultures [11]. However, we did not observe any substantial loss of axons in brain and spinal cord of presyptomatic p150^Glued^ cKO mice. Instead, we found a substantial augmentation of acetylated α-tubulin in neural tissues, including the axons of SMNs. It has been well documented that the acetylation of α-tubulin improves the stability of microtubules [36]. Therefore, the increased acetylation of α-tubulin may serve as a compensatory mechanism to maintain the integrity of microtubules in the absence of p150^Glued^. It will be interesting to delineate the signaling pathways leading to the increased α-tubulin acetylation in p150^Glued^ cKO neurons.

In addition to maintaining microtubule stability, p150^Glued^ is also proposed as a key player in uploading various cargos to the dynein motor complex in the axon terminals before being transported to the soma [34]. For example, live imaging studies show p150^Glued^ captures the cargos and loads to the microtubule plus ends for the minus-end-directed transport [37]. Accordingly, we found a lack of p150^Glued^ caused substantial disruption of NMJ structures in p150^Glued^ cKO mice. Moreover, our studies suggest that p150^Glued^ also plays a role in uploading postsynaptic glutamate receptors to dynein-mediated transport in dendrites. As results, p150^Glued^ depletion led to increased accumulation of glutamate receptors in the postsynaptic sites, which makes the p150^Glued^ cKO neurons more vulnerable to glutamate stimulation, suggesting increased excitotoxicity may contribute to the eventual loss of SMNs in p150^Glued^ cKO mice. Future studies will be needed to investigate whether the disease-causative mutations also make neurons subject to excitotoxicity.

The dynein/dynactin complex is critical for organelle transport from cell periphery to the perinuclear sites along the microtubule networks, such as the centripetal movement of autophagosomes, lysosomes and endosomes [2]. It has been shown in the previous studies that overexpressing the motor neuron disease–causative G59S mutant p150^Glued^ impairs the autophagosome and lysosome pathways [1, 30]. In line with these earlier findings, we observed similar defects in abnormal accumulation of autophagosome and lysosome proteins in p150^Glued^-lacking SMNs. The disruption of autophagosome and lysosome-mediated protein turnover thereby could also lead to the SMN loss in aging.

While in this study we focused on the roles of p150^Glued^ in CSMNs and SMNs, the floxed MTBD of p150^Glued^ can also be removed from the other neuron and cell types at different time points based on the spatial and temporal expression pattern of Cre DNA recombinase. The availability of p150^Glued^ cKO mice therefore provides a useful tool to study the functional significance of the CAP-Gly domain of p150^Glued^ in vivo.

## Conclusions

Multiple missense mutations in the CAP-Gly domain of dynactin p150^Glued^ have been linked to lower motor neuron disease, and other degenerative neurological disorders. These disease-causative mutations seem to weaken the microtubule binding affinity of p150^Glued^. However, the functional significance of p150^Glued^ has not been critically evaluated in vivo. In the present study, we generated and characterized a line of p150^Glued^ cKO mice to critically evaluate the roles of CAP-Gly domain-containing dynactin p150^Glued^ in postnatal neurons. The depletion of p150^Glued^ caused selective spinal motor neuron degeneration and movement impairments in aged mice. Substantial elevation of acetylated α-tubulin, proteins involved in autophagosome and lysosome pathways, and glutamate receptors were observed in the cKO neurons, while higher glutamate receptor levels were correlated with increase susceptibility of spinal neurons to excitotoxicity. Increased excitotoxicity may underlie a common pathogenic mechanism of p150^Glued^ mutations in diverse neurodegenerative diseases.

## Additional files

Additional file 1: **Figure S1.** Distribution of CRE in the brain and spinal cord of *Thy1-Cre* mice and depletion of p150^Glued^ in adult cKO mice. Figure S2. No apparent loss of midbrain dopaminergic neurons, striatal neurons, cerebellar granule cells and Purkinje cells in aged *Dctn1*^LoxP/LoxP^; *Thy1-Cre* mice. (DOCX 4180 kb)
