# Supplementary material for: Genetic ablation of dynactin p150Glued in postnatal neurons causes preferential degeneration of spinal motor neurons in aged mice
Source: Mol Neurodegener. 2018 Mar 1;13:10. doi: 10.1186/s13024-018-0242-z (PMC5831668; doi:10.1186/s13024-018-0242-z)
Supplement: Supplementary file 1 — Figure S1. Distribution of CRE in the brain and spinal cord of Thy1-Cre mice and depletion of p150Glued in adult cKO mice. Figure S2. No apparent loss of midbrain dopaminergic neurons, striatal neurons, cerebellar granule cells and Purkinje cells in aged Dctn1LoxP/LoxP; Thy1-Cre mice. (DOCX 4180 kb) [file 13024_2018_242_MOESM1_ESM.docx]

**Genetic ablation of dynactin p150^Glued^ in postnatal neurons causes preferential degeneration of spinal motor neurons in aged mice**

Jia Yu^1, 2^, Chen Lai^2,^ ^#^, Hoon Shim^2, ^^, Chengsong Xie^2^, Lixin Sun^2^, Cai-Xia Long^2, &^, Jinhui Ding^3^, Yan Li^4^, and Huaibin Cai^2, *^

**Additional File 1: Figure S1, S2**

**Figure S1**


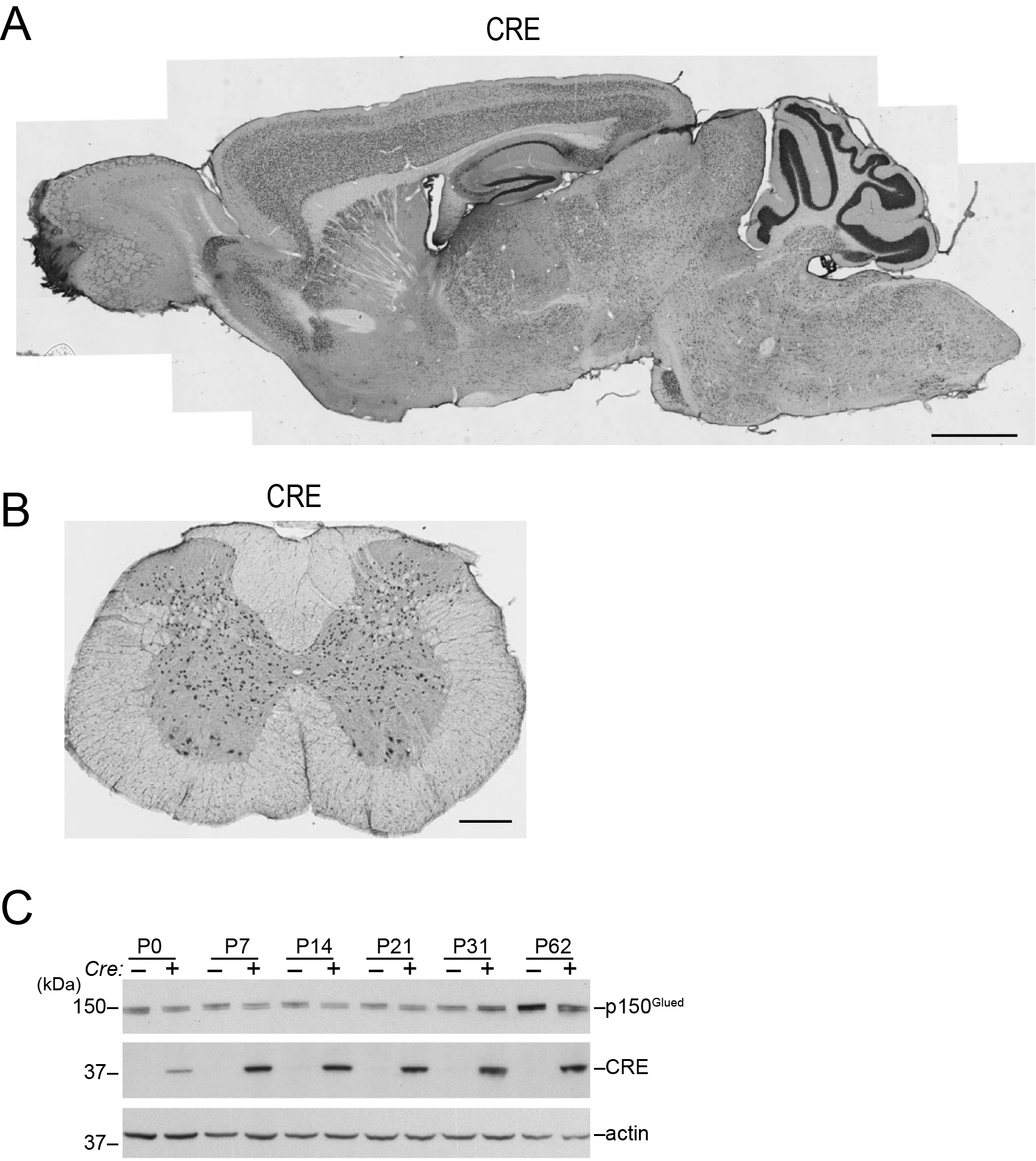


**Fig. S1 Distribution of CRE in the brain and spinal cord of *Thy1-Cre* mice and depletion of p150^Glued^ in adult cKO mice.** (A, B) Representative images show CRE staining in the sagittal brain sections (A) and coronal spinal cord sections (B) of 3-month-old *Thy1-Cre* mice. Scale bars: 2000 μm (A), 500 μm (B). (C) Western blots show p150^Glued^ and CRE levels in the brain homogenates of *Dctn1*^LoxP/LoxP^ (*Cre* –) and *Dctn1*^LoxP/LoxP^; *Thy1-Cre* (*Cre* +) mice from P0 (postnatal day 0) to P62. Actin was used as the loading control.

**Figure S2**


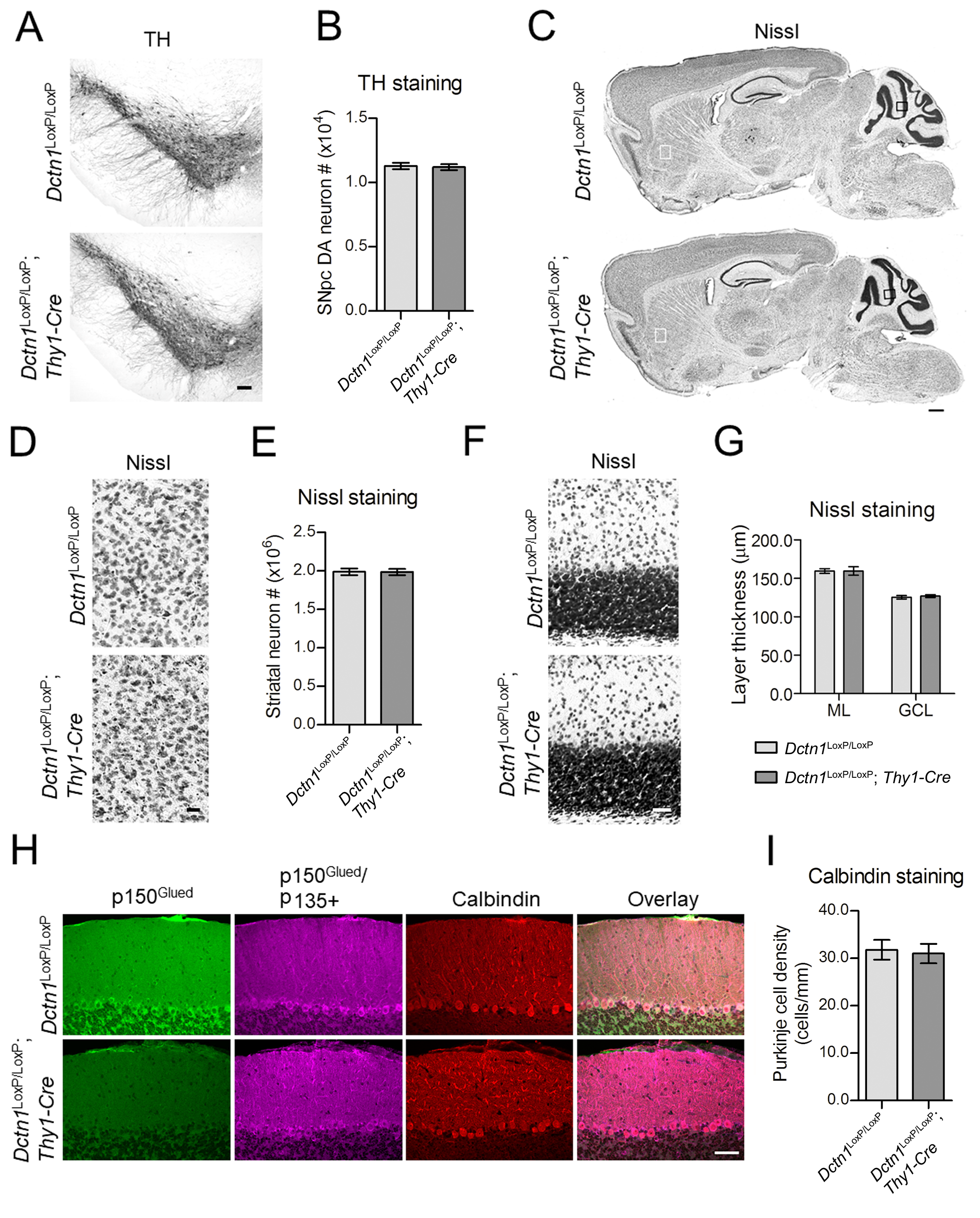


**Fig. S2 No apparent loss of midbrain dopaminergic neurons, striatal neurons, cerebellar granule cells and Purkinje cells in aged** ***Dctn1*^LoxP/LoxP^; *Thy1-Cre* mice.** (A) Representative images show TH staining of midbrain coronal sections of 18-month-old *Dctn1*^LoxP/LoxP^ and *Dctn1*^LoxP/LoxP^; *Thy1-Cre* mice. Scale bar: 100 μm. (B) Unbiased stereological estimation of the number of TH-positive dopaminergic (DA) neurons in substantia nigra pars compata (SNpc) of 18-month-old *Dctn1*^LoxP/LoxP^ and *Dctn1*^LoxP/LoxP^; *Thy1-Cre* mice (n = 6 per genotype). Data were presented as mean ± SEM. Unpaired t-test showed no statistical significance (*p* > 0.05). (C, D, F) Representative images show Nissl staining of sagittal sections of 18-month-old *Dctn1*^LoxP/LoxP^ and *Dctn1*^LoxP/LoxP^; *Thy1-Cre* mice. Scale bar: 500 μm (C, low-magnification images of whole brain, with boxed areas indicating fields shown in D and F); 50 μm (D and F, high-magnification images of striatum and cerebellum respectively). (E) Unbiased stereological estimation of the number of Nissl-stained neurons in striatum of 18-month-old *Dctn1*^LoxP/LoxP^ and *Dctn1*^LoxP/LoxP^; *Thy1-Cre* mice (n = 6 per genotype). Data were presented as mean ± SEM. Unpaired t-test showed no statistical significance (*p* > 0.05). (G) Quantification of mean thickness of molecular layer (ML) and granule cell layer (GCL) in cerebellar lobule VI of 18-month-old *Dctn1*^LoxP/LoxP^ and *Dctn1*^LoxP/LoxP^; *Thy1-Cre* mice (n = 6 mice per genotype and 9 sections per mouse). Data were presented as mean ± SEM. Unpaired t-test showed no statistical significance (ML, *p* > 0.05; GCL, *p* > 0.05). (H) Representative images show p150^Glued^ (green), p150^Glued^/135+ (purple) and Calbindin (Purkinje cell marker, red) staining in sagittal cerebellar sections of 18-month-old *Dctn1*^LoxP/LoxP^ and *Dctn1*^LoxP/LoxP^; *Thy1-Cre* mice. Scale bar: 50 μm. (I) Quantification of Purkinje cell density in cerebellar lobule VI of 18-month-old *Dctn1*^LoxP/LoxP^ and *Dctn1*^LoxP/LoxP^; *Thy1-Cre* mice (n = 6 mice per genotype and 9 sections per mouse). Data were presented as mean ± SEM. Unpaired t-test showed no statistical significance (*p* > 0.05).
